# Stroke-related ipsilateral hemiparesis: a single-centre case series and literature review

**DOI:** 10.1093/esj/aakag071

**Published:** 2026-07-06

**Authors:** François Guillaume Fenter, Davide Strambo, Alex Vicino, Elisabeth Dirren, Vincent Dunet, Patrik Michel

**Affiliations:** Stroke Center, Neurology Service, Department of Clinical Neurosciences, Lausanne University Hospital and University of Lausanne, Lausanne 1011, Switzerland; Stroke Center, Neurology Service, Department of Clinical Neurosciences, Lausanne University Hospital and University of Lausanne, Lausanne 1011, Switzerland; Stroke Center, Neurology Service, Department of Clinical Neurosciences, Lausanne University Hospital and University of Lausanne, Lausanne 1011, Switzerland; Stroke Center, Neurology Service, Geneva University Hospital, Geneva 1211, Switzerland; Neuroradiology Unit, Service of Diagnostic and Interventional Radiology, Department of Medical Radiology, Lausanne University Hospital and University of Lausanne, Lausanne 1011, Switzerland; Stroke Center, Neurology Service, Department of Clinical Neurosciences, Lausanne University Hospital and University of Lausanne, Lausanne 1011, Switzerland

**Keywords:** corticospinal, decussation, ipsilateral, paresis, stroke

## Abstract

**Introduction:**

Ipsilateral hemiparesis (ILH) in stroke is rare and challenges the conventional understanding of motor system organisation. We aim to describe ILH strokes from our academic stroke centre, complemented by a literature review.

**Patients and methods:**

All patients with hemiparesis and an acute ischaemic lesion on neuroimaging were selected from 2003 to 2023 in our stroke registry. Ipsilateral hemiparesis cases were identified, and all their investigations were reviewed, including MRI tractography, functional MRI and transcranial magnetic stimulation (if performed). We also performed a review of the literature of ILH cases in ischaemic and haemorrhagic strokes. We compared selected cases with patients with contralateral hemiparesis in our local registry.

**Results:**

Among 2433 acute ischaemic stroke patients with hemiparesis and unilateral, MRI-confirmed supratentorial lesions, we identified 4 ILH cases (0.16%). All 4 showed normal corticospinal tract (CST) decussation on neuroimaging, while functional MRI revealed bilateral motor activation in at least 1 upper limb. Our literature review identified 61 additional ILH cases meeting inclusion criteria. In exploratory analyses of the assembled dataset, ILH appeared more frequently reported in younger patients and those with prior stroke. Additional investigations were consistent with multiple potential mechanisms, including CST reorganisation, bilateral motor representation and CST anatomical variation.

**Conclusion:**

Ipsilateral hemiparesis is a rare manifestation of acute stroke that appeared more frequently reported among younger patients and those with prior strokes. However, these findings should be interpreted cautiously given the heterogeneous nature of the data sources. Some ILH may involve corticospinal reorganisation, potentially highlighting the importance of neuroplasticity in post-stroke motor control.

## Graphical Abstract

**Graphical Abstract:**
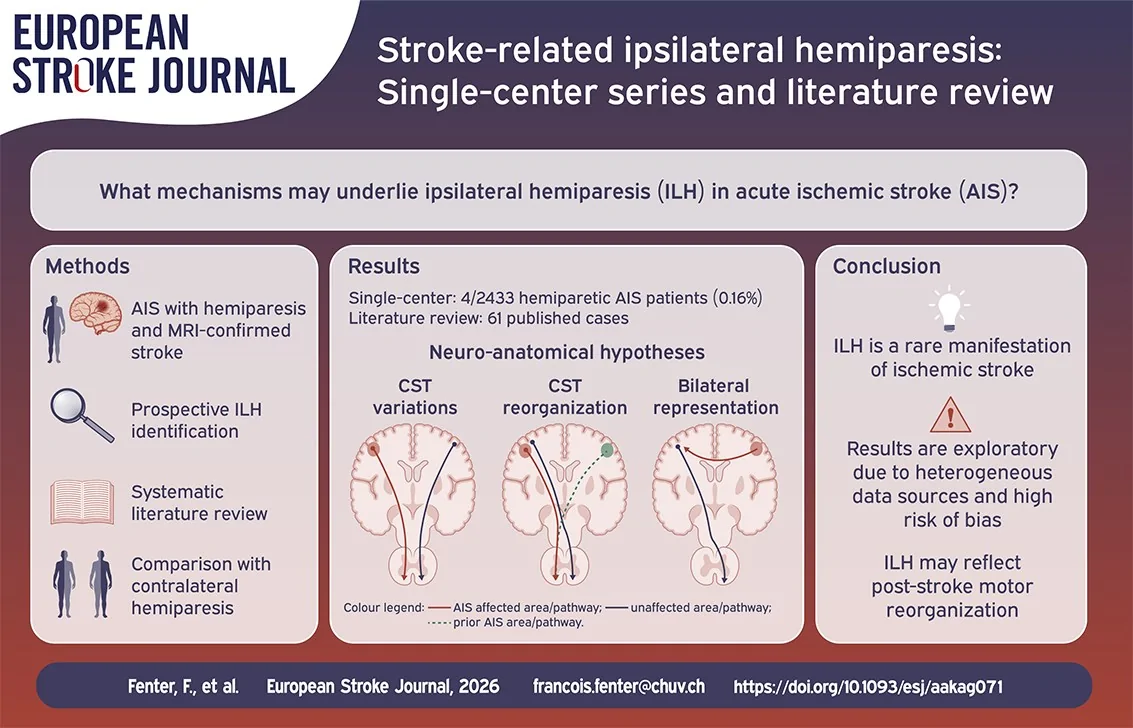

## Introduction

The corticospinal tract (CST) is the principal pathway mediating voluntary motor control. In acute stroke, motor deficits typically occur contralateral to the lesion due to the decussation of CST fibres at the medullary pyramids. This clinicoradiological relationship forms a fundamental principle guiding the neurological localisation and diagnostic evaluation of acute stroke.

Rarely, however, patients present with motor deficits on the same side as the cerebral lesion. Ipsilateral hemiparesis (ILH) challenges conventional neuroanatomical expectations and may lead to diagnostic uncertainty, particularly in the hyperacute stroke setting where treatment decisions must be made rapidly. In a previous large single-centre cohort, ILH was reported in only 0.17% of acute ischaemic strokes (AIS).^1^

Several neuroanatomical explanations have been proposed. These include congenital absence of CST decussation, such as in rare developmental disorders including ROBO3-related horizontal gaze palsy with progressive scoliosis (HGPPS),^2^ anatomical variation with partial corticospinal crossing,^3^ functional recruitment of secondary motor networks and post-lesional reorganisation of corticospinal pathways after a previous stroke.^4–6^

The aim of this study was to better characterise ILH and its neuroanatomical correlates. In this quality assurance study, we combined a prospective single-centre cohort from the Acute STroke Registry and Analysis of Lausanne (ASTRAL) with a systematic review of published cases. Our objectives were to estimate the frequency of ILH, compare its clinical characteristics with typical contralateral hemiparesis and explore potential neuroanatomical mechanisms using advanced neuroimaging and neurophysiological data.

Although individual mechanisms of ILH have previously been described in isolated case reports and small case series, the frequency of ILH, its clinical characteristics and the relative contribution of different neuroanatomical mechanisms remain poorly understood. Furthermore, comparative data against patients with typical contralateral hemiparesis are scarce.

## Methods

### Single-centre cohort from Lausanne

Patients with unilateral motor symptoms and acute ipsilateral ischaemic lesions on MRI were identified prospectively by the authors between 2003 and 2023 among all AIS patients admitted within 24 h of stroke onset to our academic stroke centre. The ASTRAL prospectively collects epidemiological, clinical, laboratory and multimodal brain imaging data of AIS patients in the Centre Hospitalier Universitaire Vaudois (CHUV).^7^

The patients were initially examined by neurologists or neurologists in training for clinical findings and stroke severity using the NIHSS score. We selected patients with acute unilateral motor symptoms and/or signs and a unilateral new cerebral ischaemic lesion shown above the medullary decussation on acute or subacute MRI within 7 days. Additional long-tract or cognitive symptoms were acceptable. We did not include patients without corticospinal symptoms (such as aphasia, hemihypoaesthesia or hemiataxia only) which are more difficult to ascertain/reproduce. We also excluded patients with ILH where an acute ischaemic lesion was only seen on CT-based imaging, given that small contralateral ischaemic lesions could be missed with this technique.

We estimated the frequency of ILH by dividing the ILH due to AIS by all AIS admitted during the same observation period using the ASTRAL registry and fulfilling the same criteria (unilateral MRI-proven lesion, contralateral hemiparesis).

For the identified ILH patients, we reviewed demographic and clinical data. Additional diagnostic evaluations were performed when considered clinically necessary, including MRI tractography, functional MRI (fMRI) and transcranial magnetic stimulation (TMS).

### Imaging

A senior neuroradiologist independently reviewed all our patients’ neuroimaging. In the acute phase all patients underwent either perfusion CT (PCT) or MRI, as previously reported.^8^ In patients with ILH according to clinical and acute imaging findings (2 had PCT, 1 MRI and 1 both CT and MRI), we subsequently performed fMRI as part of their clinical work-up. Follow-up MRI was performed after a median delay of 10 days (range 7–60) and included diffusion tensor imaging (DTI) and blood oxygen level–dependent (BOLD) fMRI during a finger-tapping task. MRI was performed on Siemens scanners (one 1.5 T and three 3 T systems). Diffusion tensor imaging slice thickness ranged from 1.6 to 3.0 mm, while BOLD-fMRI slice thickness was 3.0 mm. Diffusion tensor imaging was used to reconstruct CST tractography and assess decussation. Detailed MRI acquisition and post-processing methods are provided in the Supplementary Material.

### Transcranial magnetic stimulation

Transcranial magnetic stimulation was used to assess CST functional organisation. Transcranial magnetic stimulation with intensity above motor threshold was applied to the dorso-lateral motor cortex with a focal coil while recording both *abductor pollicis brevis* muscles. Higher amplitude of the ipsilateral motor-evoked potential (MEP) indicates an uncrossed CST.^9^

### Cases from the literature review

A systematic literature review was conducted using the PRISMA-compliant strategy to identify additional ILH cases in ischaemic and haemorrhagic stroke and reported in accordance with the PRISMA 2020 guidelines; the completed PRISMA 2020 checklist is provided in Supplementary Table 6. We searched Medline, PubMed and Cochrane databases using the terms: “stroke” AND “ipsilateral” AND (“paresis” OR “motor” OR “corticospinal” OR “pyramidal” OR “decussation” OR “crossing”), without language restrictions. Articles not written in the Latin alphabet could not be assessed and were therefore excluded. Reference lists of relevant articles were also screened for additional cases. The systematic review focused on case reports and small case series. We identified 61 cases from 20 published studies, comprising predominantly case reports and small case series.^1^^,^^4^^,^^6^^,^^10–26^

We selected reports of ILH defined as ipsilateral corticospinal motor symptoms in patients with MRI-confirmed acute ischaemic or haemorrhagic strokes located above the medullary decussation. Haemorrhagic strokes were included in the descriptive review to capture the broader spectrum of reported ILH, but excluded from exploratory analyses to align with the ischaemic ASTRAL control population and reduce heterogeneity in pathophysiology and outcome comparisons. To align with our local cohort, we included only patients with stroke diagnosed within 24 h of symptom onset and excluded subacute or chronic presentations. We excluded cases of ILH due to other pathophysiological mechanisms than stroke (eg, surgery, MS, oncology) and cases with acute bilateral strokes.

Due to incomplete reporting in the literature cases, several analyses are expected to be limited by missing data, particularly regarding precise onset-to-hospital delays, initial neurological assessment, acute treatments, etiological investigations and clinical outcomes (see Tables S3 and S4 for summary of data completeness). When not explicitly reported, admission NIHSS scores were retrospectively reconstructed from published clinical descriptions. Similarly, clinical short-term outcomes were estimated by reconstructing an mRS at discharge whenever possible; long-term follow-up was rarely reported; therefore, it was not analysed. Stroke localisation was determined by reviewing our own and published MRI images, or else from the description in the text of the publications. Given the heterogeneity and non-comparability of data sources, all comparative analyses were considered exploratory and hypothesis-generating.

### Proposed neuroanatomical framework for ILH

To assess neuroanatomical correlates of ILH, we collected data on tractography, fMRI and TMS in our and the published cases, if performed. Building on the Inatomi types I–IIa classification and incorporating additional patterns based on fMRI and tractography findings from our observations,^1^ we aimed to create a hypothesis-generating neuroanatomical framework for ILH. In addition, we distinguished ILH from “pseudo-ILH,” defined as cases in which an ipsilateral clinical deficit could not be adequately explained by the lesion alone and an alternative neurological mechanism was considered more likely (eg, contralateral ischaemia, seizure, migraine or mass effect). We then attempted to classify each local and literature-derived case into one of these categories based on all available data.

### Exploratory analysis

We combined our local and literature-identified cases for an exploratory comparison of patient and stroke characteristics. For exploratory comparative purposes, we created a control group of all ASTRAL patients during the same observation period with hemiparesis and MRI-confirmed unilateral contralateral acute stroke above the medullary decussation. As ILH cases were derived from both a prospective registry and published case reports, whereas controls originated exclusively from the ASTRAL registry, the resulting populations are inherently heterogeneous and not directly comparable.

To align the ILH cohort with the ASTRAL control group and focus on unexplained forms of ILH, we excluded haemorrhagic strokes, clinical TIA and cases with clearly documented absence of CST decussation on tractography from the exploratory analysis.

Comparisons were performed using both univariate and multivariable analyses. For univariate comparisons, we used the Wilcoxon rank-sum test for continuous variables and Fisher’s exact or chi-squared tests for categorical variables, depending on sample size.

The multivariable model for exploratory clinical comparisons of ILH as the dependent variable with controls included age, sex, admission NIHSS, stroke side, lesion depth (deep vs superficial) and history of prior stroke. Handedness and prestroke disability were not included because they were infrequently reported among literature-derived ILH cases. Functional outcome was assessed descriptively because of substantial missing data in literature-derived cases.

Year of inclusion was not modelled explicitly due to the small number of ILH cases and the limited comparability of the literature-derived cases. These comparisons do not constitute a formal case–control design, as cases and controls were derived from non-comparable populations and are presented for exploratory and hypothesis-generating purposes only.

All statistical analyses were conducted using R software (version 4.2.3; R Core Team, Vienna, Austria).^27^

### Ethical considerations and data sharing

The ASTRAL registry is approved by the local institutional authorities. Data were anonymised prior to analysis in accordance with Swiss regulations governing quality assurance projects. According to national regulations, ethics committee approval and individual consent were not required for anonymised quality assurance studies (see Declarations).

## Results

### Single-centre experience

**Figure 1:**
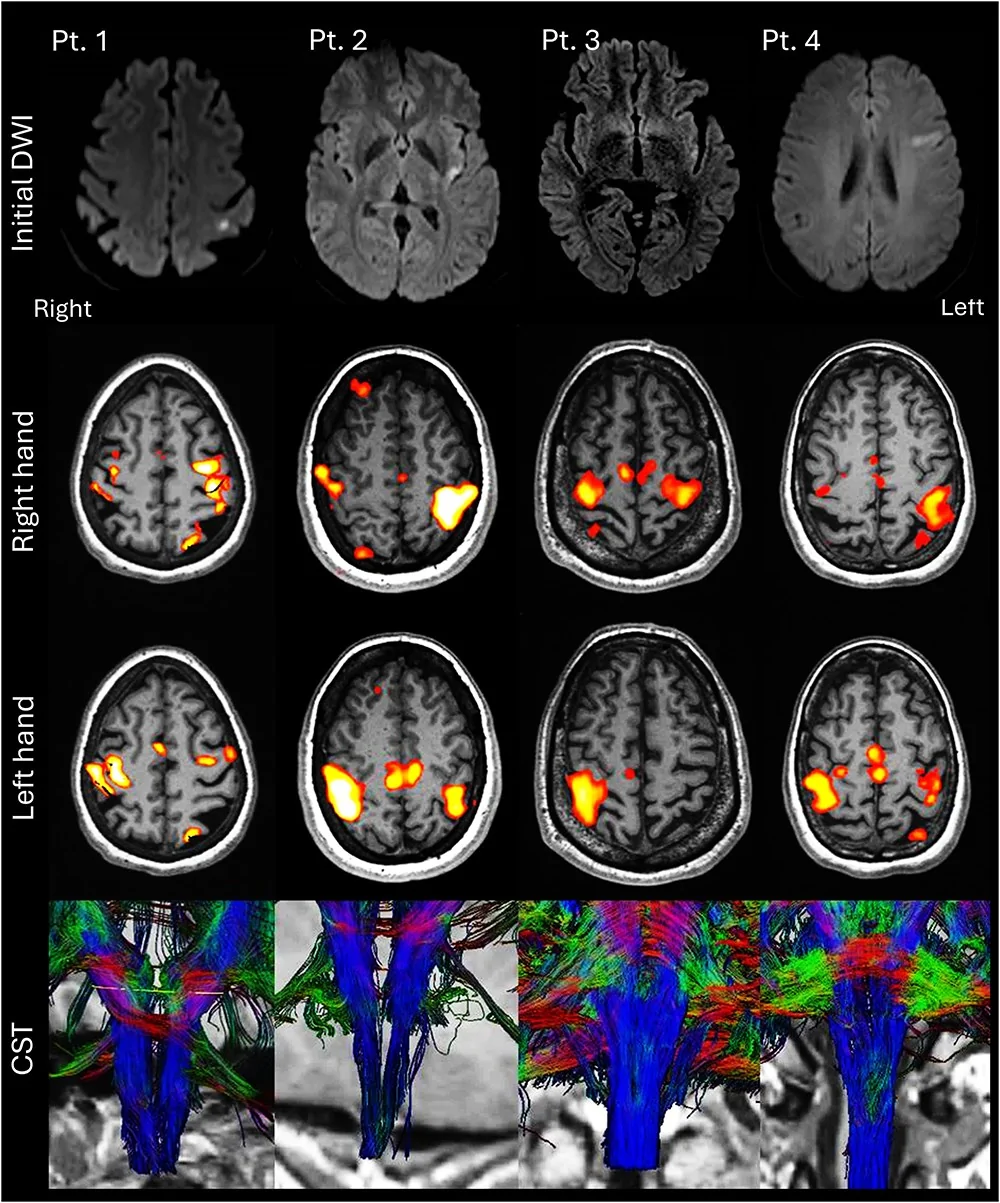
(Results, single-centre experience): Neuroimaging findings in the 4 patients with ipsilateral hemiparesis. Top row: DWI demonstrating acute ischaemic lesions in the left MCA territory in all 4 patients. Middle rows: Functional MRI (BOLD) activation maps during right- and left-hand finger tapping tasks showing predominantly bilateral cortical motor activation, including activation of the ipsilateral hemisphere relative to the moving limb. Bottom row: DTI tractography demonstrating physiological decussation of the CSTs in all patients. Abbreviations: BOLD = blood oxygen level–dependent; CST = corticospinal tract; DTI = diffusion tensor imaging.

Four patients from our institution met the inclusion criteria over a 21-year period. The estimated frequency of ILH among MRI-confirmed ischaemic stroke patients with hemiparesis was therefore 0.16% (4/2433). The main clinical and imaging characteristics are summarised in Table 1 and Figure 1, respectively.

**Table 1:** (Results, single-centre experience): Clinical and imaging characteristics of the 4 patients with ILH from our centre.

	Pat 1	Pat 2	Pat 3	Pat 4
**Demographics**	F/55y/R	F/31/R	M/54/R	F/43/L
**Vascular risk factors**	HBP, HL, smoking, obesity	HL, migraine	HBP, HL, smoking	Smoking, migraine
**Previous contralateral AIS**	Yes	No	No	No
**NIHSS, clinical syndrome**	2, left hemiparesis	5, aphasia and left hemiparesis	1, aphasia and left hemiparesis	3, left hemiparesis
**Stroke mech**	LAA	ESUS	LAA	PFO
**Lesion localisation on MRI**	Left MCA	Left MCA	Left MCA	Left MCA
**Acute treatment**	No, spontaneous resolution of symptoms	IVT after 115 min of onset	No, spontaneous improvement of symptoms	IVT after 210 min of onset
**fMRI**	Bilateral activation of post-central parietal gyrus at left upper limb motor activation	Bilateral activation of post-central parietal gyrus at right upper limb motor activation	Bilateral activation of post-central parietal gyrus at right upper limb motor activation	Bilateral activation of post-central parietal gyrus at left upper limb motor activation
**Tractography**	Physiological crossing of CST	Physiological crossing of CST	Physiological crossing of CST	Physiological crossing of CST
**TMS**	Not done (refusal)	Bilateral motor responses on stimulation on both sides	Not done (death of patient)	Physiological crossing

Abbreviations: AIS = acute ischaemic stroke; CST = cortico-spinal tract; ESUS = embolic stroke of undetermined source; F = female; fMRI = functional MRI; HBP = high blood pressure; HL = hyperlipidaemia; ILH = ipsilateral hemiparesis; IVT = intravenous thrombolysis; L = left; LAA = large-artery atherosclerosis; M = male; PFO = patent foramen ovale; R = right; TMS = transcranial magnetic stimulation.

#### Patient 1

A 55-year-old right-handed woman with vascular risk factors presented with transient left arm paresis and paraesthesia lasting 15 min. The deficit was mild (NIHSS 2 at presentation). Initial CT was normal, while day 2 MRI revealed an acute ischaemic lesion in the left postcentral gyrus. Etiological work-up was unrevealing except for a patent foramen ovale. She remained asymptomatic at 6 months (mRS 0). Tractography demonstrated normal CST decussation. Functional MRI performed on day 7 showed bilateral precentral activation during hand movement with supplementary motor area and cerebellar coactivation.

#### Patient 2

A 31-year-old right-handed woman with dyslipidaemia and migraine with aura presented with aphasia and left hemiparesis (NIHSS 5). MRI demonstrated a left M2 MCA occlusion with corresponding hypoperfusion. Intravenous thrombolysis was administered 115 min after symptom onset. Etiological investigations were unrevealing. At 6 months she had minimal residual deficit (mRS 1). Tractography showed physiological CST decussation. Functional MRI performed at 2 months demonstrated bilateral precentral activation during left-hand movement, with physiological contralateral activation for the right hand. Transcranial magnetic stimulation elicited bilateral motor responses.

#### Patient 3

A 54-year-old right-handed man with vascular risk factors presented with left facial paresis, left hemiparesis and speech disturbance. Symptoms largely resolved within 45 min except for mild facial paresis (NIHSS 1). MRI confirmed an acute left MCA territory infarct with M2 occlusion and severe ipsilateral carotid stenosis with floating thrombus. Carotid endarterectomy was performed. Twelve days later he developed a fatal intracerebral haemorrhage attributed to reperfusion syndrome. Tractography demonstrated normal CST decussation, while fMRI showed bilateral motor activation during right-hand movement.

#### Patient 4

A 43-year-old right-handed woman with migraine with aura and tobacco use presented with aphasia and left-sided sensorimotor deficit (NIHSS 3). MRI demonstrated an acute ischaemic lesion in the left frontal cortex. Intravenous thrombolysis was administered 210 min after symptom onset. Work-up revealed a patent foramen ovale, which was later closed. At 6 months she had minimal deficit (mRS 1). Tractography showed physiological CST decussation. Functional MRI performed on day 9 demonstrated bilateral activation during left-hand movement with contralateral activation for the right hand. Transcranial magnetic stimulation responses were physiological.

### Literature review

**Figure 2:**
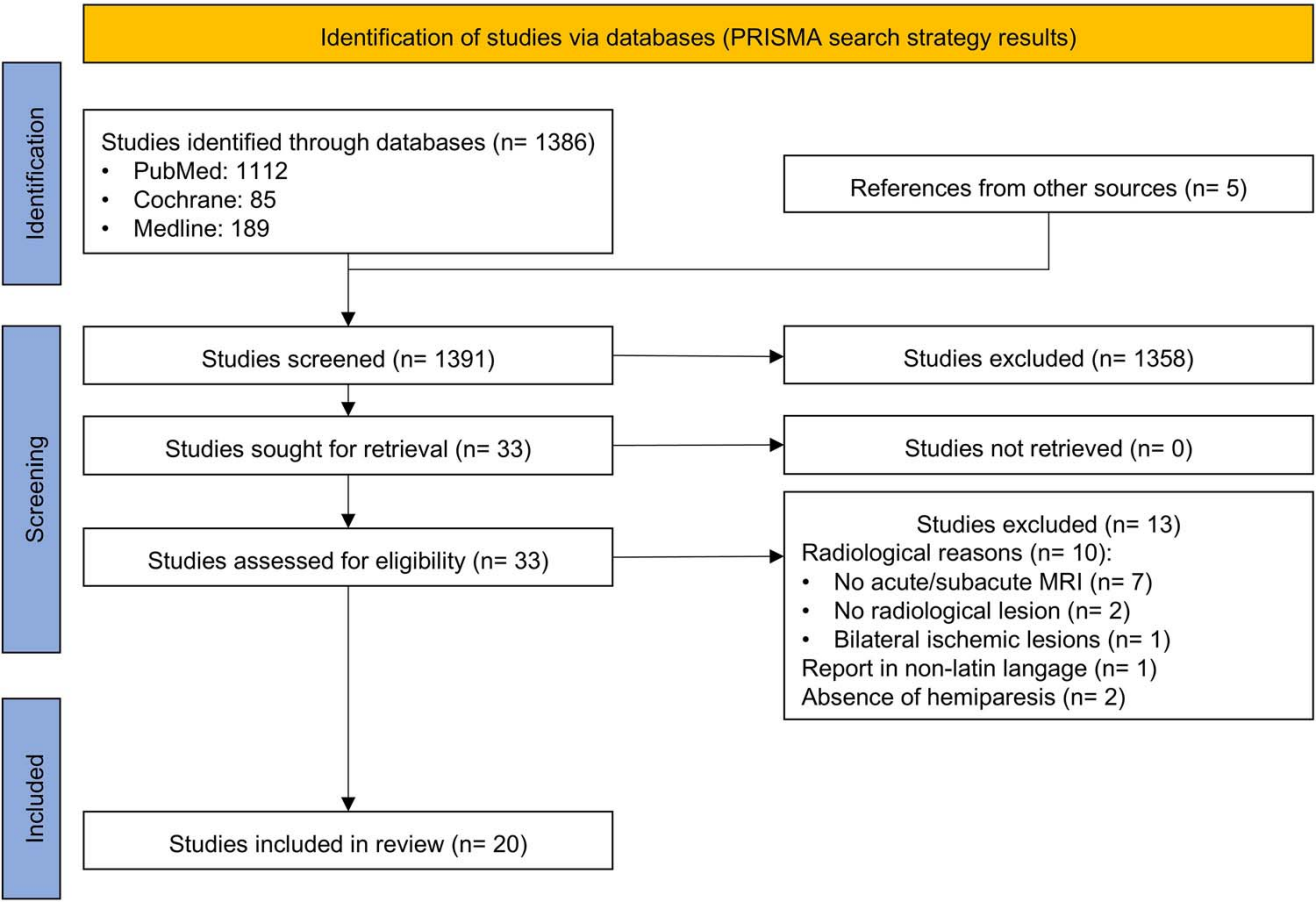
(Results, literature review): PRISMA 2020 flow diagram of study selection, December 2024. Abbreviation: PRISMA = Preferred Reporting Items for Systematic Reviews and Meta-Analyses.

We identified 61 cases from 20 studies in the literature (Figure 2). Together with our 4 local cases, this yielded a total of 65 ILH patients, including 18 fMRI studies, 14 TMS examinations and 14 tractography assessments (Table S1). Of the 65 identified ILH cases, 5 haemorrhagic strokes, 1 clinical transient ischaemic attack and 3 cases with clearly documented absence of corticospinal tract decussation on tractography were excluded from the comparative analyses, leaving 56 patients for exploratory analysis. However, these cases were retained in the overall descriptive review of ILH and contributed to the description of neuroanatomical correlates and the proposed mechanistic framework.

### Complementary exams in all ILH patients

Among the 14 tractographies, 6 revealed a complete absence of decussation, of which 2 were explained by a ROBO3 mutation. Three tractographies reported partial CST decussation, and 5 were described as physiological (Table S4).

Among the 18 fMRI studies, 15 showed bilateral activation involving at least the paretic limb (of which 4 had physiological TMS), 2 showed ipsilateral activation of at least 1 limb, 1 had physiological activation pattern.

Among the 14 TMS, 6 had bilateral motor responses on stimulation of at least 1 limb, 8 had physiological MEPs.

Results from local fMRI and tractographies are illustrated in Figure 1, and detailed descriptions of the fMRI activation patterns and arterial findings are provided in Supplementary Table 2.

### Proposed neuroanatomical framework for ILH (types I–IV)

**Figure 3:**
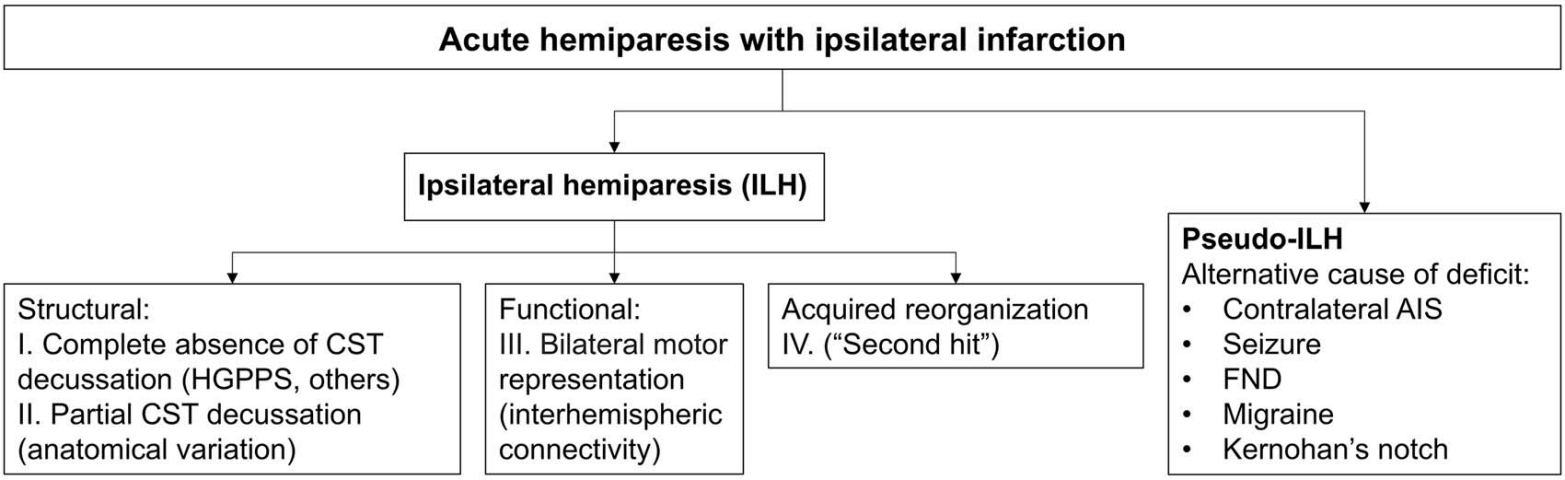
(Results, exploratory analyses): Exploratory multivariate logistic regression model showing admission characteristics associated with ILH. Results expressed as ORs and 95% CIs. The x-axis is shown on logarithmic scale. Age was modelled per 10-year increase. The model included 31 ILH patients and 2147 controls (complete-case analysis). Continuous variables are shown as median (IQR). Abbreviations: ILH = ipsilateral hemiparesis; OR = odds ratio.

Using data from neuroimaging (pre-existing lesions, fMRI and tractography) and TMS in our 4 local cases and the published cases, we propose a hypothesis-generating framework of hemiparesis with ipsilateral infarction (ILH; types I–IV), consisting of the 3 categories from Inatomi and adding a category of bilateral cortical representation suggesting interhemispheric connectivity.^1^ Ipsilateral hemiparesis refers to motor symptoms caused solely by an ipsilateral AIS, whereas pseudo-ILH involves an ipsilateral lesion coinciding with another process responsible for the clinical motor deficit. This framework should be considered conceptual and requires validation in future studies. The 4 ILH types are described below and illustrated in Figure 3; the classification of individual cases is provided in Table S5.

Type I—Absence of CST crossing (congenital): complete absence of CST decussation on tractography, as in ROBO3-related HGPPS and other rare midline malformations (Joubert, Möbius, agenesis of corpus callosum, Dandy–Walker).^28^

Type II—Bilateral CST innervation without prior stroke: preserved CST anatomy with bilateral functional activation on fMRI and TMS, suggesting inter-individual variation in corticospinal organisation.

Type III—Bilateral cortical representation via interhemispheric connectivity: normal CST anatomy on tractography and physiological TMS with bilateral activation on fMRI, suggesting involvement of associative motor areas and transcallosal pathways.

Type IV—Motor pathways reorganisation after prior contralateral stroke or “second-hit”: ipsilateral motor control secondary to post-stroke contralesional reorganisation, with bilateral or ipsilateral TMS responses and bilateral or contralesional activation on fMRI.

Pseudo-ILH refers to cases where an ipsilateral lesion coincides with another process causing the motor deficit. Possible explanations include undetected contralateral stroke, stroke mimics such as migraine or seizure, or mechanical compression of the CST such as Kernohan’s notch phenomenon.

In our local series, patient 1 had a history of previous contralateral stroke and was classified as a possible “second-hit” ILH case (type IV), although pseudo-ILH could not be excluded given the transient nature of symptoms and the timing of imaging. Patients 2 and 3 were considered possible cases of CST anatomical variation not detected on tractography (type II). Because of the bilateral fMRI activation pattern in the presence of normal tractography, patient 4 was considered a possible interhemispheric connectivity variant (type III).

Among 65 cases, we identified 38 possible “second-hit” cases (type IV), 7 cases of complete absence of decussation (type I), including 2 cases of HGPPS, 4 cases of CST anatomical variation or partial decussation (type II) and 4 cases of interhemispheric connectivity variant (type III). Twelve cases could not be classified due to missing information.

### Exploratory analyses

Exploratory comparisons between AIS patients with ILH (*n* = 56) and controls (*n* = 2429) showed that the ILH group appeared younger (median age 61 vs 72 years), had a similar sex distribution and a higher prevalence of previous stroke (71.4% vs 18.3%), while admission stroke severity (NIHSS) was similar between groups (Table 2). Among ILH patients with previous ischaemic stroke, the prior lesion was contralateral to the new infarct in 85% of cases. Ipsilateral hemiparesis patients also tended to have higher pre-stroke mRS scores. Neglect was significantly less frequent in ILH patients than in controls (0% vs 26.4%). There were otherwise no evident differences in non-motor symptoms. Only 14% of ILH patients received acute revascularisation therapy compared with 57% of controls; this difference could not be adjusted for onset-to-hospital delay because these data were frequently missing in the reported literature cases. Functional disability at discharge appeared similar in both groups, although outcome data were incompletely reported in many literature-derived cases.

**Table 2:** (Results, exploratory analyses): Univariate comparison of population of AIS with ILH, with the control group from ASTRAL with MRI-confirmed contralateral hemiparesis. Data are given as median and IQR, or absolute numbers and percentages. Denominators vary across variables because of incomplete reporting in published cases. Percentages are calculated using available data; denominators vary due to missing information.

Variable	Control group	AIS with ILH	Significance
	*n* = 2429	*n* = 56	*P*-value
**Demographics and stroke severity**			
**Age (years)**	72.3 (22.3, *n* = 2426)	61.0 (16.75, *n* = 34)	<.001
**Female sex**	1028/2426 (42.4%)	13/34 (38.2%)	.756
**Left-handedness**	198/2181 (9.1%)	4/34 (11.8%)	.545
**Pre-stroke Rankin**	0 (2, *n* = 2428)	1.5 (2, *n* = 22)	.002
**Previous stroke**	445/2429 (18.3%)	40/56 (71.4%)	<.001
**Discharge Rankin**	2 (3, *n* = 2327)	1 (1.75, *n* = 14)	.057
**Admission NIHSS**	6 (9, *n* = 2425)	8 (4, *n* = 53)	.469
**Vascular risk factors**			
**Hypertension**	224/370 (60.5%)	13/16 (81.2%)	.119
**Diabetes**	54/361 (15.0%)	5/14 (35.7%)	.086
**Dyslipidaemia**	253/368 (68.8%)	10/15 (66.7%)	1.000
**Smoking**	178/363 (49%)	7/15 (46.7%)	1.000
**Atrial fibrillation**	58/372 (15.6%)	4/14 (28.6%)	.255
**Migraine history**	7/110 (6.4%)	2/6 (33.3%)	.068
**Obesity**	420/2311 (18.2%)	2/10 (20%)	1.000
**Associated symptoms**			
**Sensory**	1130/2424 (46.6%)	12/27 (44.4%)	.975
**Aphasia**	820/2429 (33.8%)	5/24 (20.8%)	.264
**Neglect**	641/2426 (26.4%)	0/19 (0%)	.006
**Dysarthria**	1365/2429 (56.2%)	7/14 (50%)	.845
**Cerebellar**	548/2429 (22.6%)	1/8 (12.5%)	.692
**Acute management**			
**Acute revascularisation treatment**	1385/2423 (57.2%)	2/14 (14.3%)	.002
**Vascular territory**			
**PCA**	171/2425 (7.1%)	0/31 (0%)	.165
**MCA**	1741/2425 (71.8%)	15/19 (78.9%)	.614
**ACA**	51/2425 (2.1%)	1/31 (3.2%)	.487
**Deep (vs superficial)**	1428/2154 (66.3%)	38/50 (76%)	.198
**Stroke side (left)**	1256/2429 (51.7%)	30/56 (53.6%)	.888

Abbreviations: AIS = acute ischaemic stroke; ACA = anterior cerebral artery; ILH = ipsilateral hemiparesis; PCA = posterior cerebral artery.

**Figure 4:**
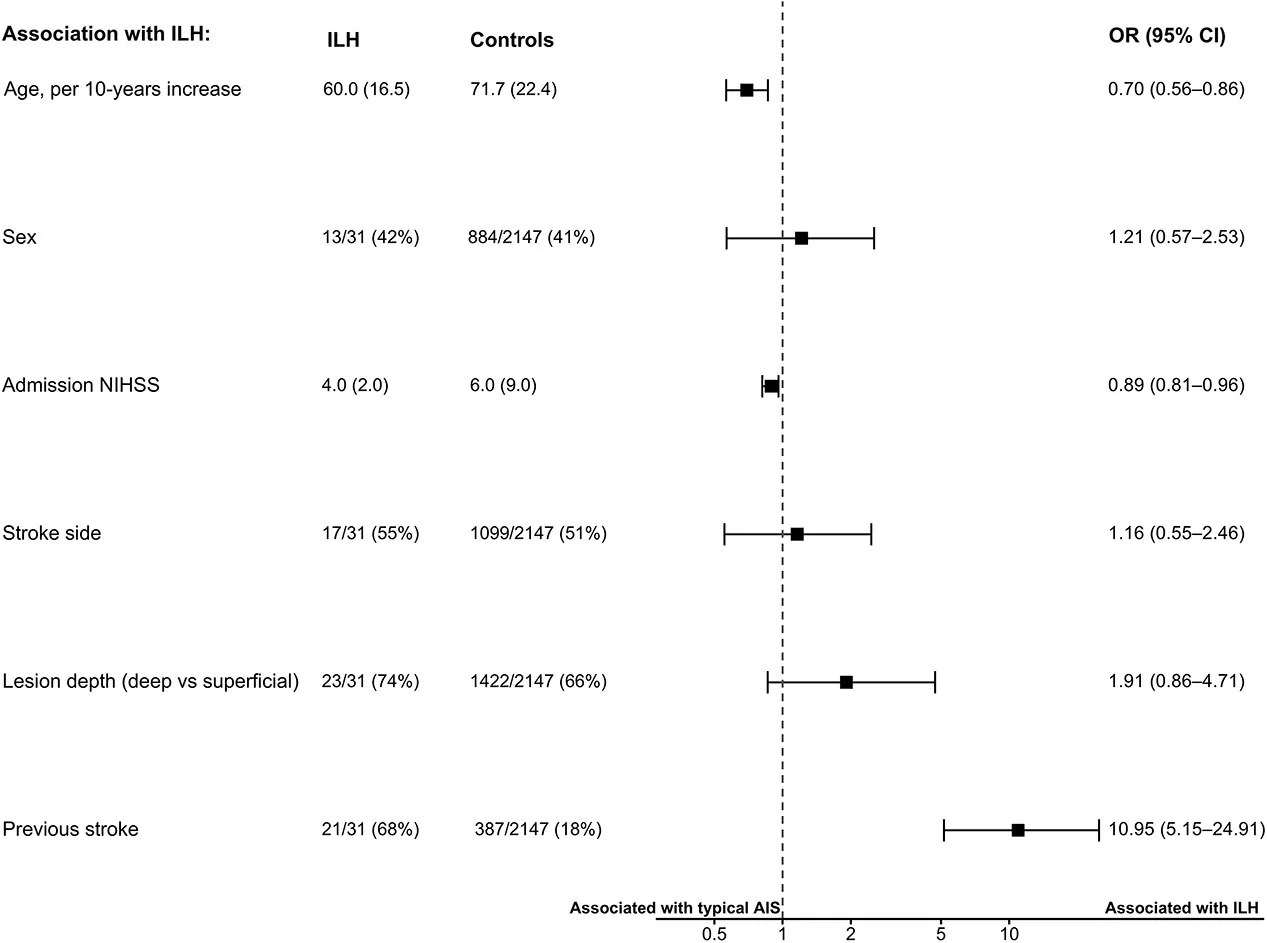
(Results, proposed neuroanatomical framework for ILH): Proposed diagnostic and mechanistic framework for ILH with ipsilateral radiological infarction. Acute hemiparesis with ipsilateral infarction can be classified into ILH and pseudo-ILH. ILH may arise from 3 main mechanisms: (1) structural variation of the CST, including complete (type I) or partial (type II) absence of decussation; (2) bilateral motor representation (type III), suggesting interhemispheric connectivity and (3) acquired reorganisation following prior contralateral stroke (“second-hit”; type IV). In contrast, pseudo-ILH refers to cases in which an alternative neurological cause is more likely (eg, contralateral ischaemic stroke, seizure, functional neurological disorder, migraine or Kernohan’s notch phenomenon). Abbreviations: AIS = acute ischaemic stroke; CST = corticospinal tract; FND = functional neurological disorder; HGPPS = horizontal gaze palsy with progressive scoliosis; ILH = ipsilateral hemiparesis.

In exploratory multivariable analyses (complete-case analysis; 31 ILH patients and 2147 controls), ILH appeared to be associated with younger age, lower admission NIHSS and prior stroke (Figure 4). Lesion depth (deep vs superficial) was not significantly associated with ILH. Neglect was not included in the multivariable analysis because no ILH patient presented neglect, resulting in complete separation. These findings should be interpreted with caution given the heterogeneous and non-comparable nature of the data sources.

## Discussion

Tractography findings were heterogeneous. Six patients had complete absence of CST crossing (including 2 genetically confirmed ROBO3-related HGPPS), 5 were normal. Three had described partial decussation, 2 among bilateral fMRI activation patterns. Functional MRI patterns ranged from purely contralateral to bilateral or ipsilateral activation, and TMS revealed bilateral activation in a substantial subset of 6/14. The most frequently identified neuroanatomical correlate was type IV (38 cases), followed by type I (7 cases), types II and III (4 cases each).

### Complementary exams interpretation

These findings should be interpreted cautiously, as ancillary investigations were available in only a subset of patients and were performed at variable time points after stroke onset.

In the 14 ILH patients where tractography was performed, about two-thirds displayed completely or partially absent CST decussation (64%). Although tractography is useful to detect such variations, its sensitivity to do so is limited.^29^ Moreover, partial decussation is a subjective description without clear definitions, and macroscopic fibre pathway descriptions are highly method-dependent.^30^

Among the 18 patients with fMRI, the majority showed bilateral activation of at least 1 limb, which may support the hypothesis of bilateral cortical motor representation. Functional MRI is widely used to assess post-lesional network reorganisation, but similar activation patterns may arise from different connectivity mechanisms.^31–34^ Ipsilateral M1 activation varies across healthy individuals depending on demographic factors (age, handedness),^35^ task characteristics and methodological factors.^36–40^ Bilateral motor activation is also frequently observed after stroke, particularly in the subacute or chronic phases and in patients with more severe deficits, with variability depending on lesion characteristics, imaging quality and timing of assessment.^31^^,^^41^^,^^42^ Consequently, post-stroke fMRI findings may not reliably reflect pre-existing CST organisation. Nevertheless, fMRI may detect ILH mechanisms involving secondary motor areas that would not be identified by tractography or TMS.

Among the 14 patients who underwent TMS, we found that the majority exhibited normal physiological responses (8/14). Five patients showed bilateral activation of at least 1 limb and only 1 patient had ipsilateral motor activation. This may be consistent with a type II neuroanatomical correlate of ILH. Transcranial magnetic stimulation is useful to identify bilateral and infra-radiological CST anomalies. Bilateral MEPs in healthy subjects have also been described,^43^ and the technique remains nonetheless examiner-dependent and subject to methodological variability.

A substantial proportion of patients exhibited bilateral motor cortex activation on fMRI. However, most of these cases showed no corresponding abnormalities on TMS (4/7) or tractographic evidence of CST non-decussation (4/5). These discrepancies may reflect the involvement of secondary motor areas, variability of individual functional somatotopy or the timing of assessment in the subacute phase of stroke, as discussed above.

Pseudo-ILH cases were not specifically evaluated here, as this analysis focused on neuroanatomical correlates in cases considered compatible with ILH.

### Proposed neuroanatomical framework for ILH

Integrating the available neuroimaging, neurophysiological and clinical findings, we propose a hypothesis-generating framework comprising 4 potential neuroanatomical mechanisms of ILH while also distinguishing true ILH from pseudo-ILH. This framework should not be viewed as a definitive classification but rather as a conceptual model to organise reported mechanisms and to facilitate interpretation of this rare clinicoradiological presentation. Future studies using standardised imaging and neurophysiological assessments are needed to validate these proposed categories.

Type I corresponds to congenital absence of CST decussation and represents the most straightforward explanation of ILH. Although rare, this mechanism is well established in ROBO3-related HGPPS and other developmental disorders affecting midline crossing pathways. The presence of 6 cases with complete absence of decussation supports the validity of this mechanism but suggests that it accounts for only a minority of ILH presentations.

Type II likely reflects inter-individual variation in CST anatomy,^44^ and may explain the presence of discrete ipsilateral symptoms in classical motor strokes.^45^ The small number of identified cases and the limited sensitivity of tractography make this mechanism difficult to confirm. Some patients classified as type II may in fact represent subtle variants of type IV reorganisation or partially decussating CSTs not detected by available imaging techniques.

Type III likely reflects bilateral cortical motor representation mediated by interhemispheric connectivity despite preserved corticospinal tract anatomy. Reported bilateral motor symptoms in epileptic discharges involving the secondary motor area could point towards this hypothesis.^46^ The frequent bilateral activation observed on fMRI with normal CST tractography (4/5) and contralateral MEPs on TMS (4/7) could be consistent with this hypothesis, although such findings could also result from other acute or subacute connectivity mechanisms.

Type IV likely reflects post-stroke motor pathway reorganisation following a prior contralateral lesion and was the most frequently identified pattern in our assembled dataset. The association between prior contralateral stroke and ILH, together with the predominance of contralateral antecedent lesions, may be consistent with a “second-hit” model in which pre-existing motor reorganisation unmasks ipsilateral motor deficits after a subsequent stroke. However, because advanced investigations were not systematically available, this remains speculative. Moreover, the phenomenon of contralesional reorganisation after a stroke is dynamic in time, region-specific, age-dependent and patient-dependent, with potential facilitating or hindering effect on motor recovery.^47^^,^^48^ In some patients classified as type II or III, subtle or previously unrecognised CST alterations cannot be excluded, and a reorganisation mechanism similar to type IV may have contributed. Prior stroke alone is clearly insufficient to explain ILH, given that recurrent stroke is common whereas ILH remains exceptionally rare. Additional factors, such as the location of the antecedent lesion, individual variability in motor network organisation and the extent of post-stroke reorganisation, are likely required.

### Exploratory findings

In exploratory comparisons with the ASTRAL control cohort, ILH cases appeared younger and more frequently had a history of previous stroke, often contralateral to the new ischaemic lesion.

We did not identify a clear explanation for the younger age observed in ILH patients. Publication bias in the literature is possible, but we also observed this lower age distribution in our local ILH cases when compared to our own control group (although the very small number of 4 local ILH cases severely limits interpretation). This may reflect greater neuroplasticity at younger age or persistence of ipsilateral corticospinal projections during development.^3^^,^^49^ However, the small number of cases limits interpretation.

Lower admission NIHSS emerged only in the complete-case multivariable analysis and was not observed in crude comparisons. This finding should therefore be interpreted cautiously, as it may reflect the effects of case selection and model adjustment rather than a true biological association.

The higher prevalence of previous stroke in ILH patients may be consistent with the predominance of type IV cases in our proposed framework. These findings should nevertheless be interpreted with caution given the exploratory nature of the analyses and the limitations discussed above.

### Limitations

This study combines prospectively collected registry data with cases extracted from the literature, resulting in a highly heterogeneous dataset with non-standardised clinical documentation, imaging protocols and timing of assessments. The inclusion of published cases also exposes the study to substantial selection and publication bias. The comparison with ASTRAL controls does not constitute a formal case–control design and should be interpreted as exploratory comparisons only, as cases and controls were derived from non-comparable populations with different inclusion mechanisms and incomplete adjustment for confounding. Key variables, including NIHSS and short-term functional outcomes, were partly reconstructed from published reports, introducing a risk of misclassification. Missing data further limited analyses and may have introduced additional bias. Furthermore, the rarity of ILH resulted in a limited number of cases available for comparative analyses, reducing statistical power and increasing uncertainty around estimated associations. Finally, the long inclusion period (2003–2023) introduced temporal heterogeneity in imaging techniques and stroke management, which was not explicitly accounted for in the analyses.

Advanced imaging modalities (fMRI, TMS, tractography) were performed in only a subset of patients, at variable time points, without standardised protocols or comparison groups, limiting their interpretability. Because DTI and fMRI were performed at variable intervals after stroke onset, the observed findings may reflect both pre-existing neuroanatomical organisation and post-stroke functional reorganisation. Although all included cases had MRI-confirmed unilateral lesions, the presence of a very small contralateral ischaemic lesion below the detection threshold of MRI cannot be excluded.

## Conclusion

Acute hemiparesis with ipsilateral stroke lesions is a rare presentation that may reflect a spectrum of underlying neuroanatomical mechanisms. In our assembled dataset, ILH cases appeared younger and more frequently had a history of prior contralateral stroke; however, these observations should be interpreted with caution given the heterogeneous and non-comparable nature of the data sources. Pseudo-ILH may arise from the coexistence of an incidental lesion and a separate neurological process responsible for the motor deficit. Improved recognition of these entities, along with careful interpretation of advanced imaging findings, may help reduce diagnostic uncertainty and avoid unnecessary treatment escalation in acute stroke care.

We do not advocate routine use of advanced neurophysiological or functional imaging investigations in all cases of suspected ILH. Their role currently remains exploratory and may be considered selectively when clarification of the underlying mechanism is clinically relevant.

## Supplementary Material

Ipsilateral_suppl_final_aakag071

## Data Availability

The data that support the findings of this study are available upon request from the corresponding author. The data are not publicly available due to privacy or ethical restrictions.
