# Supplementary material for: Stroke-related ipsilateral haemiparesis: a single-centre case series and literature review
Source: Eur Stroke J. 2026 Jul 6;11(7):aakag071. doi: 10.1093/esj/aakag071 (PMC13335639; doi:10.1093/esj/aakag071)
Supplement: Ipsilateral_suppl_final_aakag071 [file ipsilateral_suppl_final_aakag071.docx]

**Supplementary Material to:**

**“Stroke-related ipsilateral hemiparesis: a single-center case series and literature review”**

Fenter F^1^, Strambo D^1^, Vicino A^1^, Dirren E^2^, Dunet V^3^, Michel P^1^

# 1 Stroke Center, Neurology Service, Department of Clinical Neurosciences, Lausanne University Hospital and University of Lausanne, 1011 Lausanne, Switzerland

# 2 Stroke Center, Neurology Service, Geneva University Hospital, 1211 Geneva, Switzerland

3 Neuroradiology Unit, Service of Diagnostic and Interventional Radiology, Department of Medical Radiology, Lausanne University Hospital and University of Lausanne, 1011 Lausanne, Switzerland

**Supplementary Methods: MRI acquisition**

Follow-up MRI were performed within a median delay of 10 days (min-max: 7-60 d) and included 3D T1 weighted magnetization prepared rapid gradient echo (MP-RAGE), diffusion tensor imaging (DTI), and blood oxygenation level-dependent (BOLD) sequences during a two-step right then left index finger tapping task. DTI was post-processed with Syngovia MR Neuro 3D software (Version XA 60, Siemens, Erlangen, Germany) to obtain MR-tractography and to assess the corticospinal tract (CST) for decussation. BOLD activation maps were generated based on General Linear Model (GLM) analysis and exported to the institutional picture archiving and communication system (PACS) for combined assessment with the tractography.

**Supplementary Results: Detailed case descriptions**

*Patient 1:*

A 55-year-old right-handed woman with multiple risk factors presented with transient left upper limb paresis and paresthesia of acute onset. Symptoms resolved spontaneously within 15 minutes. After an initial, normal head-CT with perfusion, day-2 brain MRI revealed an acute ischemic lesion in the left post-central gyrus. Workup, including transthoracic and transesophageal echocardiography, was unrevealing except for a patent foramen ovale. On antiplatelet therapy and risk factor control, she had no recurrence and no residual deficit at six months (mRS 0). MRI-tractography showed no corticospinal tract abnormality. fMRI performed at day 7 demonstrated bilateral precentral motor activation for both hands, with bilateral coactivation of the supplementary motor area (SMA) and cerebellum during right-hand movement.

*Patient 2:*

A 31-year-old right-handed woman with dyslipidaemia and migraine with visual aura presented with acute aphasia and left hemiparesis (NIHSS of 5). Day 0 MRI revealed a left M2 middle cerebral artery (MCA) occlusion with hypoperfusion in the corresponding territory. She received intravenous thrombolysis 115 minutes after symptom onset. Work-up was unrevealing and the patient remained recurrence-free over six months (mRS 1) on antiplatelet and lipid-lowering therapy. MRI-tractography showed physiological corticospinal decussation. fMRI performed at 2 months revealed bilateral precentral activation during left-hand movement and left-lateralized activation (i.e., physiological) for right-hand movement. TMS elicited bilateral motor responses, with higher response rates from the left hemisphere (70%) than the right (40%).

*Patient 3:*

A 54-year-old right-handed man with multiple risk factors presented with left facial paresis, left hemiparesis, and speech impairment. Symptoms resolved spontaneously within 45 minutes except for the left central facial paralysis (NIHSS 1). Hyperacute CT showed no acute cerebral lesion but a hyperdense sign of the second portion of the left MCA. Day 1 MRI showed an acute left MCA territory DWI lesion and a left MCA occlusion (M2). Carotid Doppler and MRI revealed a left 80–90% atheromatous stenosis of the internal carotid artery with a floating thrombus. He underwent left carotid endarterectomy on day five. On day 12 after the stroke (7 days after the intervention), he developed a right hemiplegia and impaired consciousness, attributed to a left hemispheric intraparenchymal haemorrhage due to a reperfusion syndrome. After discussion with his next of kin, a palliative attitude was adopted and the patient died on day 13. MRI-tractography on day 11 after the ischemic stroke confirmed physiological corticospinal decussation and the fMRI revealed bilateral precentral activation for the right hand and right-lateralized precentral activation for the left hand, accompanied by bilateral SMA and cerebellar coactivation during right-hand movement.

*Patient 4:*

A 43-year-old right-handed woman with tobacco use (8 pack-years) and migraine with visual aura presented with aphasia and left-sided sensorimotor deficits affecting both upper and lower limbs (NIHSS = 3). Day 0 MRI revealed an acute ischemic lesion in the left frontal cortex. She received intravenous thrombolysis 210 minutes after symptom onset. Workup revealed a patent foramen ovale. Two months later, she experienced a recurrence of left hemifacial dysesthesia without new ischemic lesions on MRI. At six months, mRS was 1. The patent foramen ovale was subsequently closed. MRI-tractography showed physiological corticospinal crossing. fMRI performed at day 9 demonstrated bilateral precentral activation during left hand movement and purely contralateral activation for the right hand. Motor evoked potentials were only recorded on the hand contralateral to the TMS cortical stimulation, with no recordable ipsilateral potential, indicating a normal corticospinal tract decussation.

**Supplementary Results: Excluded studies**

Twelve articles including 14 patients were excluded because they did not meet the inclusion criteria: there was no acute MRI reported in the publications from Kudo, Schneider, Fischer, Lagger, Cuatico, Yamamoto, Hosokawa, Terakawa, and Manal. ^51-58^ The patient from Xu had bilateral strokes on MRI, the case from Kajtazi did not have hemiparesis, and the report of two patients by Chen was written in Chinese only. ^49, 59, 60^

Among these excluded cases, we would like to mention 1 additional case of absent CST crossing (Manal), ^58^ but no other clinical abnormalities. We also mention 2 cases of probable HGPPS with ipsilateral activation on TMS (Hosokawa, Terakawa). ^56, 57^

**Supplementary Tables**

**Supplementary Table 1** (Results, Literature review): Availability of neuroimaging and neurophysiological data in cases of ILH identified through the literature review, without local cases.

| Author | Date | N | MRI | fMRI | TMS | Tractography |
| --- | --- | --- | --- | --- | --- | --- |
| Ago ^34^ | 2003 | 1 | 1 | 1 | 0 | 0 |
| Song ^35^ | 2005 | 2 | 2 | 2 | 0 | 0 |
| Kang ^36^ | 2010 | 1 | 1 | 0 | 1 | 0 |
| Ng ^37^ | 2011 | 1 | 1 | 0 | 0 | 1 |
| Alurkar ^38^ | 2012 | 1 | 1 | 0 | 0 | 1 |
| Saada^4^ | 2013 | 2 | 2 | 0 | 0 | 0 |
| Jang ^39^ | 2013 | 1 | 1 | 0 | 0 | 1 |
| Yamada ^40^ | 2015 | 1 | 1 | 0 | 0 | 1 |
| Kobayashi ^41^ | 2015 | 1 | 1 | 0 | 1 | 0 |
| Hebant ^42^ | 2016 | 2 | 2 | 0 | 0 | 0 |
| Inatomi ^1^ | 2016 | 14 | 14 | 9 | 7 | 0 |
| Patra ^6^ | 2018 | 1 | 1 | 1 | 0 | 1 |
| Tan ^43^ | 2019 | 22 | 22 | 0 | 0 | 0 |
| Yang ^44^ | 2020 | 1 | 1 | 0 | 0 | 1 |
| Reddy ^45^ | 2021 | 1 | 1 | 0 | 0 | 1 |
| Tan ^46^ | 2021 | 1 | 1 | 1 | 1 | 1 |
| Zhou ^47^ | 2021 | 1 | 1 | 0 | 0 | 1 |
| Mala ^48^ | 2023 | 1 | 1 | 0 | 0 | 1 |
| Kajtazi ^49^ | 2023 | 5 | 5 | 0 | 1 | 0 |
| Porey ^50^ | 2023 | 1 | 1 | 0 | 1 | 0 |
| Total |  | 61 | 61 | 14 | 12 | 10 |

**Supplementary Table 2.** Description of fMRI activation and arterial findings in our four ILH patients.

| Patient | Arterial Territory | Arterial Abnormality | Precentral Motor Activation (Right /Left Hand) | SMA Coactivation (Right /Left Hand) | Cerebellar Coactivation (Right /Left Hand) |
| --- | --- | --- | --- | --- | --- |
| 1 | MCA | none | Bilateral/ Bilateral | Bilateral / Bilateral | Bilateral / Bilateral |
| 2 | MCA | M2 occlusion | Left / Bilateral | Left / Bilateral | Left / Bilateral |
| 3 | MCA | M2 stenosis | Bilateral / Right | Bilateral / Right | Bilateral/ Left |
| 4 | MCA | M3 occlusion | Left / Bilateral | Left / Bilateral | Right / Bilateral |

**Supplementary Table 3**: Summary of data of interest from the literature review available for analysis.

| Author | N | mRS t-1 | mRS | emRs | Discharge mRS | Follow-up mRS | NIHSS | eNIHSS | Discharge NIHSS |
| --- | --- | --- | --- | --- | --- | --- | --- | --- | --- |
| Ago ^34^ | 1 | 0 | 0 | 0 | 0 | 0 | 0 | 0 | 0 |
| Song ^35^ | 2 | 1 | 0 | 1 | 0 | 0 | 0 | 2 | 0 |
| Kang ^36^ | 1 | 0 | 0 | 1 | 1 | 0 | 0 | 1 | 0 |
| Ng ^37^ | 1 | 1 | 1 | 0 | 1 | 0 | 1 | 0 | 0 |
| Alurkar ^38^ | 1 | 0 | 0 | 0 | 0 | 0 | 0 | 0 | 0 |
| Saada^4^ | 2 | 0 | 0 | 2 | 2 | 0 | 0 | 2 | 0 |
| Jang ^39^ | 1 | 1 | 1 | 0 | 0 | 0 | 0 | 1 | 0 |
| Yamada ^40^ | 1 | 0 | 0 | 0 | 0 | 0 | 0 | 0 | 0 |
| Kobayashi ^41^ | 1 | 0 | 0 | 0 | 0 | 0 | 0 | 1 | 0 |
| Hebant ^42^ | 2 | 2 | 0 | 2 | 2 | 0 | 0 | 0 | 0 |
| Inatomi ^1^ | 14 | 14 | 0 | 0 | 0 | 0 | 0 | 12 | 0 |
| Patra ^6^ | 1 | 0 | 1 | 0 | 1 | 0 | 1 | 0 | 0 |
| Tan ^43^ | 22 | 0 | 1 | 0 | 1 | 1 | 0 | 1 | 0 |
| Yang ^44^ | 1 | 0 | 0 | 0 | 0 | 0 | 0 | 1 | 0 |
| Reddy ^45^ | 1 | 0 | 0 | 0 | 0 | 0 | 1 | 0 | 0 |
| Tan ^46^ | 1 | 0 | 1 | 0 | 1 | 1 | 0 | 1 | 0 |
| Zhou ^47^ | 1 | 0 | 0 | 0 | 0 | 0 | 0 | 1 | 0 |
| Mala ^48^ | 1 | 0 | 1 | 0 | 1 | 0 | 0 | 1 | 0 |
| Kajtazi ^49^ | 5 | 1 | 0 | 0 | 4 | 1 | 5 | 0 | 0 |
| Porey ^50^ | 1 | 1 | 1 | 0 | 1 | 0 | 0 | 0 | 0 |
| Total | **61** | **19** | **7** | **6** | **15** | **3** | **8** | **24** | **0** |

**Supplementary Table 4:** Results of complementary exams in ours and published cases

| Author | fMRI (motor activation,  upper limb) | TMS | Tractography | Genetic |
| --- | --- | --- | --- | --- |
| CHUV | Bilateral activation  of paretic limb | NA | Physiological | NA |
|  | Bilateral activation  of non-paretic limb | Bilateral activation | Physiological | NA |
|  | Bilateral activation  of non-paretic limb | NA | Physiological | NA |
|  | Bilateral activation  of paretic limb | Physiological | Physiological | NA |
| Ago ^34^ | Left activation  for left limb | NA | NA | NA |
| Song ^35^ | Bilateral activation  of paretic limb | NA | NA | NA |
|  | Bilateral activation  of paretic limb | NA | NA | NA |
| Kang ^36^ | NA | Bilateral ipsilateral  activation | NA | NA |
| Ng ^37^ | NA | NA | Absence of  CST crossing | ROBO3 |
| Alurkar ^38^ | NA | NA | Absence of  CST crossing | NA |
| Jang ^39^ | NA | NA | Partial CST  crossing | NA |
| Yamada ^40^ | NA | NA | Absence of  CST crossing | ROBO3 |
| Kobayashi ^41^ | NA | Physiological | NA | NA |
| Inatomi | Bilateral activation  of paretic limb | Bilateral activation of  paretic limb | NA | NA |
|  | Bilateral activation  of both limb | Physiological | NA | NA |
|  | NA | Physiological | NA | NA |
|  | NA | Physiological | NA | NA |
|  | Bilateral activation  of paretic limb | Physiological | NA | NA |
|  | Bilateral activation  of both limb | NA | NA | NA |
|  | Physiological | NA | NA | NA |
|  | Bilateral activation  of paretic limb | Physiological | NA | NA |
|  | Bilateral activation  of paretic limb | NA | NA | NA |
|  | Bilateral activation  of both limb | NA | NA | NA |
|  | Bilateral activation of  paretic limb | Bilateral activation of  healthy limb | NA | NA |
| Patra ^6^ | Predominantly ipsilateral  activation | NA | Partial CST  crossing | NA |
| Yang ^44^ | NA | NA | Absence of  CST crossing | NA |
| Reddy ^45^ | NA | NA | Absence of  CST crossing | NA |
| Tan ^46^ | Bilateral activation of  paretic limb | Bilateral activation  of both limbs | Partial CST  crossing | NA |
| Zhou ^47^ | NA | NA | Absence of  CST crossing | NA |
| Mala ^48^ | NA | NA | Physiological | NA |
| Kajtazi ^49^ | NA | Physiological | NA | NA |
| Porey ^50^ | NA | Bilateral activation  of both limbs | NA | NA |

NA = not available

**Supplementary table 5**: Classification of selected cases according to the proposed classification.

| Author | Previous contra-lateral stroke | Stroke localization  superficial & frontal lobe | fMRI (motor activation,  upper limb) | TMS | Tractography | Genetic | Proposed ILH subtype |
| --- | --- | --- | --- | --- | --- | --- | --- |
| CHUV | 1 | 0 | Bilateral activation  of paretic limb | NA | Physiological | NA | IV |
|  | 0 | 0 | Bilateral activation  of non-paretic limb | Bilateral activation | Physiological | NA | II |
|  | 0 | 0 | Bilateral activation  of non-paretic limb | NA | Physiological | NA | II |
|  | 0 | 1 | Bilateral activation  of paretic limb | Physiological | Physiological | NA | III |
| Ago ^34^ | 1 | 0 | Left activation  for left limb | NA | NA | NA | IV |
| Song ^35^ | 1 | 0 | Bilateral activation  of paretic limb | NA | NA | NA | IV |
|  | 1 | 0 | Bilateral activation  of paretic limb | NA | NA | NA | IV |
| Kang ^36^ | 0 | 0 | NA | Bilateral ipsilateral  activation | NA | NA | I |
| Ng ^37^ | 0 | 0 | NA | NA | Absence of  CST crossing | ROBO3 | I |
| Alurkar ^38^ | 0 | 0 | NA | NA | Absence of  CST crossing | NA | I |
| Saada^4^ | 1 | 0 | NA | NA | NA | NA | IV |
|  | 1 | 0 | NA | NA | NA | NA | IV |
| Jang ^39^ | 1 | 0 | NA | NA | Partial CST  crossing | NA | IV |
| Yamada ^40^ | 0 | 0 | NA | NA | Absence of  CST crossing | ROBO3 | I |
| Kobayashi ^41^ | 1 | 0 | NA | Physiological | NA | NA | IV |
| Hebant ^42^ | 1 | 0 | NA | NA | NA | NA | IV |
|  | 1 | 0 | NA | NA | NA | NA | IV |
| Inatomi ^1^ | 1 | 0 | NA | NA | NA | NA | IV |
|  | 0 | 0 | Bilateral activation  of paretic limb | Bilateral activation of  paretic limb | NA | NA | IV |
|  | 1 | 0 | Bilateral activation  of both limb | Physiological | NA | NA | IV |
|  | 0 | 0 | NA | Physiological | NA | NA | III |
|  | 1 | 0 | NA | NA | NA | NA | IV |
|  | 1 | 0 | NA | NA | NA | NA | IV |
|  | 0 | 0 | NA | Physiological | NA | NA | III |
|  | 1 | 0 | Bilateral activation  of paretic limb | Physiological | NA | NA | IV |
|  | 1 | 0 | Bilateral activation  of both limb | NA | NA | NA | IV |
|  | 1 | 0 | Physiological | NA | NA | NA | IV |
|  | 1 | 0 | Bilateral activation  of paretic limb | Physiological | NA | NA | IV |
|  | 0 | 0 | Bilateral activation  of paretic limb | NA | NA | NA | II or III |
|  | 0 | 0 | Bilateral activation  of both limb | NA | NA | NA | II or III |
|  | 1 | 0 | Bilateral activation of  paretic limb | Bilateral activation of  healthy limb | NA | NA | IV |
| Patra ^6^ | 0 | 1 | Predominantly ipsilateral  activation | NA | Partial CST  crossing | NA | II |
| Tan ^43^ | 1 | 0 | NA | NA | NA | NA | IV |
|  | 0 | 0 | NA | NA | NA | NA | Unknown |
|  | 1 | 1 | NA | NA | NA | NA | IV |
|  | 1 | 0 | NA | NA | NA | NA | IV |
|  | 1 | 0 | NA | NA | NA | NA | IV |
|  | 0 | 0 | NA | NA | NA | NA | Unknown |
|  | 1 | 0 | NA | NA | NA | NA | IV |
|  | 0 | 0 | NA | NA | NA | NA | Unknown |
|  | 1 | 0 | NA | NA | NA | NA | IV |
|  | 0 | 0 | NA | NA | NA | NA | Unknown |
|  | 0 | 0 | NA | NA | NA | NA | Unknown |
|  | 1 | 0 | NA | NA | NA | NA | IV |
|  | 1 | 0 | NA | NA | NA | NA | IV |
|  | 0 | 0 | NA | NA | NA | NA | Unknown |
|  | 1 | 0 | NA | NA | NA | NA | IV |
|  | 1 | 0 | NA | NA | NA | NA | IV |
|  | 1 | 0 | NA | NA | NA | NA | IV |
|  | 0 | 1 | NA | NA | NA | NA | III |
|  | 0 | 0 | NA | NA | NA | NA | Unknown |
|  | 1 | 0 | NA | NA | NA | NA | IV |
|  | 1 | 0 | NA | NA | NA | NA | IV |
|  | 1 | 1 | NA | NA | NA | NA | IV |
| Yang ^44^ | 0 | 0 | NA | NA | Absence of  CST crossing | NA | I |
| Reddy ^45^ | 0 | 0 | NA | NA | Absence of  CST crossing | NA | I |
| Tan ^46^ | 0 | 0 | Bilateral activation of paretic limb | Bilateral activation  of both limbs | Partial CST  crossing | NA | II |
| Zhou ^47^ | 0 | 1 | NA | NA | Absence of  CST crossing | NA | I |
| Mala ^48^ | 1 | 0 | NA | NA | Physiological | NA | IV |
| Kajtazi ^49^ | 1 | 1 | NA | Physiological | NA | NA | IV |
|  | 0 | 1 | NA | NA | NA | NA | Unknown |
|  | 0 | 0 | NA | NA | NA | NA | Unknown |
|  | 1 | 0 | NA | NA | NA | NA | IV |
|  | 0 | 0 | NA | NA | NA | NA | Unknown |
| Porey ^50^ | 1 | 0 | NA | Bilateral activation  of both limbs | NA | NA | IV |

NA = not available, Type I-IV according to proposed classification (see results).

**Supplementary table 6:** PRISMA 2020 Checklist :

| **Section** | **Item** | **PRISMA 2020 checklist item** | **Location in manuscript** |
| --- | --- | --- | --- |
| Title | 1 | The report is identified as a systematic review | Title |
| Abstract | 2 | Structured summary including background, objectives, methods, results, and conclusions | Abstract |
| Introduction | 3 | Rationale for the review in the context of existing knowledge | Background |
|  | 4 | Explicit statement of objectives | Introduction (Aims paragraph) |
| Methods | 5 | Eligibility criteria for inclusion | Methods – Literature review |
|  | 6 | Information sources and date of last search | Methods – Literature review |
|  | 7 | Search strategy and search terms | Methods – Literature review |
|  | 8 | Study selection process | Methods – Literature review |
|  | 9 | Data extraction and handling of missing data | Methods – Literature review |
|  | 10 | Definition of variables and outcomes | Methods – Analysis; Supplementary Tables |
|  | 11 | Risk of bias assessment | Methods – Analysis (ROBINS-I) |
|  | 12 | Effect measures used | Methods – Analysis |
|  | 13a | Methods of synthesis | Methods – Analysis |
|  | 13b | Statistical methods used for comparisons | Methods – Analysis |
|  | 13c | Exploration of heterogeneity | Methods – Analysis |
|  | 13d | Sensitivity analyses | Not applicable (heterogeneous rare-case literature) |
|  | 14 | Assessment of reporting bias | Not applicable (case reports and small case series) |
|  | 15 | Assessment of certainty of evidence | Not applicable (descriptive evidence base) |
| Results | 16a | Results of literature search and study selection | Results – Literature review; PRISMA flow diagram |
|  | 16b | Reasons for exclusion of full-text articles | Results – Literature review |
|  | 17 | Characteristics of included studies | Results – Literature review; Supplementary Tables |
|  | 18 | Risk of bias in included studies | Results – Limitations and risk of bias |
|  | 19 | Results of individual studies | Results – Literature review |
|  | 20 | Results of syntheses | Results – Comparative analyses |
|  | 21 | Reporting bias assessment | Not applicable |
|  | 22 | Certainty of evidence | Not applicable |
| Discussion | 23a | Interpretation of results in context of existing evidence | Discussion |
|  | 23b | Limitations of the evidence | Limitations |
|  | 23c | Limitations of the review process | Limitations |
|  | 23d | Implications for clinical practice and future research | Discussion |
| Other information | 24a | Registration information | Not registered |
|  | 24b | Protocol availability | Not applicable |
|  | 25 | Sources of funding | No external funding |
|  | 26 | Competing interests | The authors report no competing interests |
|  | 27 | Data availability | Ethical considerations and data sharing |

Several PRISMA 2020 items (14, 15, 21, 22) were not applicable because the review was based on case reports and small case series without intervention comparisons or pooled effect estimates.

**Supplementary References**

50. Porey C, Bhoi SK, Jha M, Samal P, Naik S. Recurrent stroke with ipsilateral hemiparesis: an unusual phenomenon. *Acta Neurol Belg*. 2023;123(3):1161-1164. doi:10.1007/s13760-022-02163-0

51. Kudo T, Uno T. Ipsilateral Hemiparesis Caused by Subarachnoid Hemorrhage in a Patient with a Ruptured Middle Cerebral Artery Aneurysm: A Case Report. *Neurosurgery*. 1984;15(5):727-729. doi:10.1227/00006123-198411000-00019

52. Schneider RC, Crosby E. Ipsilateral Symptoms Caused by an Arteriovenous Malformation of the Second or Supplementary Sensory Area of the Island of Reil. *Neurosurgery*. 1983;12(5):557-560. doi:10.1227/00006123-198305000-00014

53. Fisher CM. Concerning the mechanism of recovery in stroke hemiplegia. *Can J Neurol Sci*. 1992;19(1):57-63.

54. Cuatico W. The phenomenon of ipsilateral innervation. One case report. . *J Neurosurg Sci* . Published online 1979.

55. Yamamoto H, Bogousslavsky J. Ipsilateral hemiparesis and pyramidal tract abnormalities. *Neurology*. 1998;50(1):316. doi:10.1212/wnl.50.1.316

56. Hosokawa S, Tsuji S, Uozumi T, Matsunaga K, Toda K, Ota S. Ipsilateral hemiplegia caused by right internal capsule and thalamic hemorrhage. *Neurology*. 1996;46(4):1146-1149. doi:10.1212/WNL.46.4.1146

57. Terakawa H, Abe K, Nakamura M, Okazaki T, Obashi J, Yanagihara T. Ipsilateral hemiparesis after putaminal hemorrhage due to uncrossed pyramidal tract. *Neurology*. 2000;54(9):1801-1805. doi:10.1212/WNL.54.9.1801

58. Jidal M, Horache K, Fikri M, El Kettani N, Jiddane M, Touarsa F. A rare case of ispilateral hemiparesis in a patient with uncrossed pyramidal tract shown by tractography. *Radiol Case Rep*. 2024;19(8):3512-3516. doi:10.1016/j.radcr.2024.04.088

59. Xu Y, Liu L. Ipsilateral hemiparesis and contralateral lower limb paresis caused by anterior cerebral artery territory infarct. *Neurosciences*. 2016;21(3):256-259. doi:10.17712/nsj.2016.3.20150701

60. Chen YC. Ipsilateral Hemiparesis Caused by Supratentorial Stroke: Two casereports. *Rehabilitation Practice and Science*. 2012;40(4). doi:10.6315/2012.40(4)07
